# Dupilumab Failure in Treating Dermatomyositis-Associated Pruritus

**DOI:** 10.7759/cureus.28270

**Published:** 2022-08-22

**Authors:** Margaret A O'Brien, Sylvia Hsu, Alina Shevchenko, Andrea Quartey

**Affiliations:** 1 Department of Dermatology, Temple University Lewis Katz School of Medicine, Philadelphia, USA

**Keywords:** itch, il-31, dupilumab, pruritus, dermatomyositis

## Abstract

Dermatomyositis (DM) is a multisystem inflammatory condition with diverse cutaneous and systemic symptoms. Both muscle and skin involvement are common and can occur simultaneously or sequentially, or individuals can have muscle- or skin-limited disease. Skin involvement in DM can be extensive, and pruritus is one of the most problematic symptoms for the patient. Its pathophysiology is poorly understood, making management challenging for clinicians. A limited number of therapeutic agents target pruritus in DM, adding another challenge for clinicians. Previous case reports suggest dupilumab as a treatment for pruritus in DM. However, our patient experienced no relief. Our patient’s failure of dupilumab suggests that its targets, interleukin (IL)-4 and IL-13, do not play a significant role in the pruritus of DM. It is possible that targeting other small molecules in inflammatory pathways could greatly alleviate pruritus for individuals with DM. Further studies need to be conducted to extrapolate the mechanism of pruritus in DM so that individuals with DM can find more significant relief.

## Introduction

Dermatomyositis (DM) is a multisystem inflammatory condition presenting with a diverse array of cutaneous and systemic signs [1]. Its wide variety of systemic presentations often makes this disease underdiagnosed with prolonged times to diagnosis. It is estimated that DM affects approximately 1 to 6 per 100,000 adults in the United States and has a bimodal onset in childhood and adulthood [1]. Skin findings in DM can range from pathognomonic to nonspecific, including Gottron papules, Gottron sign, heliotrope rash, holster sign, and V sign. Muscle manifestations are also variable with individuals having acute or subacute onset of symmetric proximal weakness, which can be painless or asymptomatic with elevation in muscle enzymes [1]. Often, persistent pruritus is one of the most problematic symptoms for the patient. Its pathophysiology is poorly understood, therefore making its management challenging. Many of the first-line agents for skin-limited DM do not adequately treat DM-associated pruritus. Although successful mitigation of DM-associated pruritus with dupilumab was recently reported in a case report [2], the trial of dupilumab in our case did not improve the patient’s symptoms suggesting that IL-4 and IL-13 do not play a dominant role as mediators of itch in this condition.

## Case presentation

**Figure 1:**
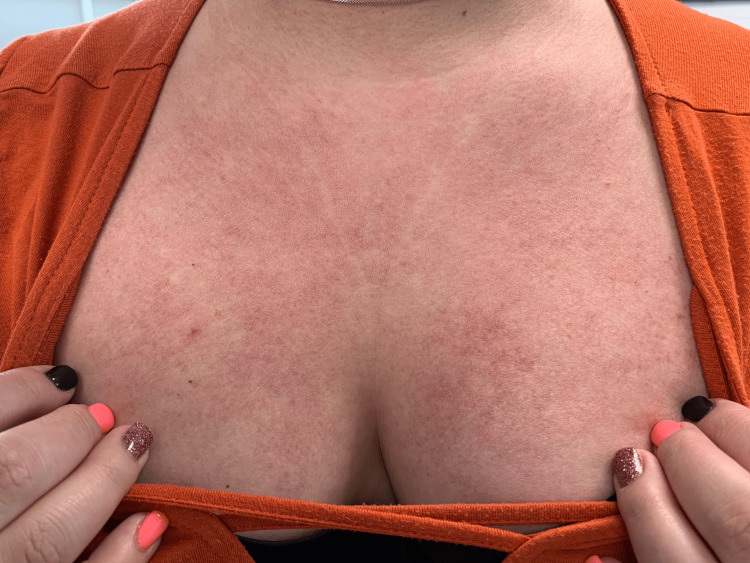
The V sign of dermatomyositis

**Figure 2:**
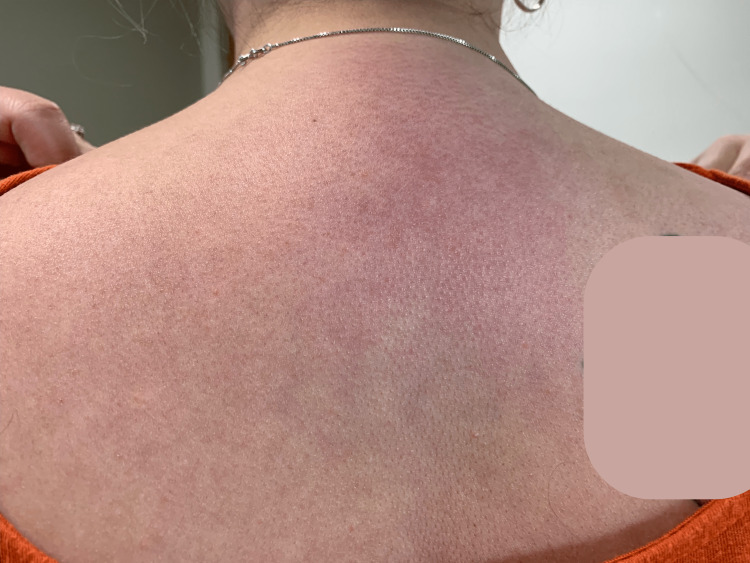
The shawl sign of dermatomyositis

A 28-year-old woman presented to our clinic with a pruritic, erythematous rash on her face, particularly her eyelids and malar cheeks, chest, and upper extremities. She had sparing of the submental neck and was noted to have bilaterally erythematous patches on her hips. Biopsy showed an interface dermatitis, consistent with our clinical diagnosis of DM. Her initial creatinine kinase (CK) was elevated at 249 U/L (normal range: 24-173 U/L), and aldolase was within the normal range at 4.4 U/L (normal range: 3.3-10.3 U/L). Skin biopsy confirmed our clinical suspicion of DM. Follow-up EMG performed by her rheumatologist was consistent with proximal myopathy. Cancer screening was negative. Despite strict photoprotection, the patient was continuously tormented by intractable pruritus on her chest, upper back, and upper lateral arms (Figures 1, 2). Since her diagnosis, the patient had tried multiple medications, including topical and oral corticosteroids, dapsone, methotrexate, azathioprine, hydroxychloroquine, and thalidomide. All of these agents had failed to provide any significant alleviation of her pruritus. The patient was then started on dupilumab 600 mg administered subcutaneously (SQ) on day 1 and then 300 mg SQ every other week. However, she reported only mild improvement in her symptoms after a trial of five months. Ultimately, dupilumab was discontinued, and the patient was switched to intravenous immunoglobulin (IVIg) 2 g/kg IV over four days per month, leading to a rapid improvement of her pruritus after the first cycle.

## Discussion

Pruritus is one of the most challenging cutaneous manifestations of DM, negatively impacting the patient’s quality of life. Dupilumab, a human monoclonal antibody that inhibits the signaling of both IL-4 and IL-13 by blocking the IL-4R⍺ subunit, has been successfully used to manage pruritus in patients with Th2-mediated inflammatory diseases such as atopic dermatitis and asthma [3,4]. A case report by Bowers and Huang recently demonstrated significant improvement of pruritus in a patient with DM treated with dupilumab [2]. Unfortunately, dupilumab did not have that effect in mitigating our patient’s pruritus, and her symptoms have only improved significantly after starting IVIg.

According to recent studies, IL-31 has been gaining more attention as a potential driver of pruritus. Specifically, Kim et al. noticed that in their study group of 191 patients with DM, those with moderate-to-severe itch showed evidence of IL-31 and IL-31 receptor alpha (IL-31RA) upregulation compared to non-lesional and healthy control skin [5]. Additional studies involving animal models have also suggested IL-31 overexpression to be associated with severe pruritus, alopecia, and skin lesions [6].

Even though dupilumab has also been shown to reduce levels of other proinflammatory cytokines, including IL-31 [7], the fact that it did not work in our case may suggest that its effect on this cytokine in DM was either insufficient or that a more complex interplay of cytokines is responsible for the mechanism of pruritus in DM, with both of these options being possible. Furthermore, our case suggests that the pathophysiology of itch in DM might not be driven by the same mediators seen in other intensely pruritic diseases, such as atopic dermatitis, and that more research and drug development are needed to better address this issue in patients with DM.

The exact mechanism of how IVIg relieves pruritus in DM is unclear. However, our patient’s successful relief after starting IVIg supports one of its proposed mechanisms of modulating the production of inflammatory cytokines [8]. IVIg has been used successfully in case reports to treat other intensely pruritic diseases such as atopic dermatitis, with subsequent reduction in inflammatory cytokines like IL-4 [9]. The complex mechanism of IVIg mirrors the complexity of DM and hopefully can give more insight into this disease's pathogenesis. More targeted agents have been described in the literature with the potential to improve pruritus through modulation of IL-31, albeit many of the studies were conducted in patients with atopic dermatitis and not DM. Nemolizumab, a humanized monoclonal antibody against IL-31RA, reportedly decreased pruritus in patients with atopic dermatitis when used with topical agents [10]. Another agent, lenabasum, an analog of tetrahydrocannabinol, has been shown to decrease levels of multiple molecules involved in DM, such as tumor necrosis factor-alpha (TNFα), interferon (IFN)-α, and IFN-β, as well as IL-31 levels [5], findings that potentially have promising results for the future management of intractable pruritus in DM.

## Conclusions

As the case reports about the efficacy of dupilumab in pruritus management in DM are inconsistent, further studies are required to elucidate the mechanism of pruritus in this condition. Additional studies in patients with DM are needed to determine the efficacy of these therapeutics in managing intractable pruritus in patients with DM. With the advent of studies exploring the role of other cytokines, itch, and novel agents targeting these molecules, there is optimism regarding the future of symptom improvement for these patients.
